# The Contribution of White Matter Diffusion and Cortical Perfusion Pathology to Vascular Cognitive Impairment: A Multimode Imaging-Based Machine Learning Study

**DOI:** 10.3389/fnagi.2021.687001

**Published:** 2021-08-06

**Authors:** Yao Wang, Peiwen Lu, Yafeng Zhan, Xiaowei Wu, Yage Qiu, Zheng Wang, Qun Xu, Yan Zhou

**Affiliations:** ^1^Department of Radiology, RenJi Hospital, School of Medicine, Shanghai Jiao Tong University, Shanghai, China; ^2^Department of Neurology, RenJi Hospital, School of Medicine, Shanghai Jiao Tong University, Shanghai, China; ^3^Center for Excellence in Brain Science and Intelligence Technology, State Key Laboratory of Neuroscience, Key Laboratory of Primate Neurobiology, Institute of Neuroscience, Chinese Academy of Sciences, Shanghai, China

**Keywords:** small vessel disease, multimode imaging, machine learning, diffusion tensor imaging, arterial spin labeling

## Abstract

Widespread impairments in white matter and cerebrovascular integrity have been consistently implicated in the pathophysiology of patients with small vessel disease (SVD). However, the neural circuit mechanisms that underlie the developing progress of clinical cognitive symptoms remain largely elusive. Here, we conducted cross-modal MRI scanning including diffusion tensor imaging and arterial spin labeling in a cohort of 113 patients with SVD, which included 74 patients with vascular mild cognitive impairment (vMCI) and 39 patients without vMCI symptoms, and hence developed multimode imaging-based machine learning models to identify markers that discriminated SVD subtypes. Diffusion and perfusion features, respectively, extracted from individual white matter and gray matter regions were used to train three sets of classifiers in a nested 10-fold fashion: diffusion-based, perfusion-based, and combined diffusion-perfusion-based classifiers. We found that the diffusion-perfusion combined classifier achieved the highest accuracy of 72.57% with leave-one-out cross-validation, with the diffusion features largely spanning the capsular lateral pathway of the cholinergic tracts, and the perfusion features mainly distributed in the frontal-subcortical-limbic areas. Furthermore, diffusion-based features within vMCI group were associated with performance on executive function tests. We demonstrated the superior accuracy of using diffusion-perfusion combined multimode imaging features for classifying vMCI subtype out of a cohort of patients with SVD. Disruption of white matter integrity might play a critical role in the progression of cognitive impairment in patients with SVD, while malregulation of coritcal perfusion needs further study.

## Introduction

Vascular dysfunction and associated cerebral damage have been identified as critical components of the pathophysiology of late-life dementia, and may constitute the predominant pathological cause of cognitive impairment in East Asia (Iadecola et al., 2019). Patients with small vessel disease (SVD) have recently been receiving increasing attention because of its high prevalence (Rosenberg et al., 2016; Wardlaw et al., 2019). SVD is generally referred to as a disorder of cerebral microvessels causing widespread physiological and structural abnormalities including subcortical lacunar infarcts, white matter hyperintensities (WMH), and microbleeds (Pantoni, 2010; Rosenberg et al., 2016). The pathogenesis of SVD has been attributed to a wide variety of pathological events including vessel occlusion, leakage of toxins, impaired vascular reactivity, decreased clearance of waste products, oligodendrocyte dysfunction, increased oxidation, and inflammation. These pathological events give rise to diverse brain lesions that are able to be detected by using different imaging modalities (Schuff et al., 2009; Duering et al., 2015; Sun et al., 2016; Duncombe et al., 2017; Muñoz Maniega et al., 2017), although the relationships between the imaged lesions and clinical symptoms remain poorly understood (Wardlaw et al., 2013, 2019). As the management of risk factors and symptom-specific treatment could help prevent the evolution of small vascular mild cognitive impairment (vMCI, the prodromal stage of vascular dementia) to vascular dementia (Seo et al., 2010), there is an urgent need to identify imaging-based biomarkers for early diagnosis and monitoring disease progression.

Aggregated evidence obtained from case-control designs has demonstrated associations between cognitive decline in patients with SVD and widespread cerebral impairments of various kinds such as cerebral perfusion and WM integrity (O'Sullivan et al., 2001, 2004; Tuladhar et al., 2015; Shi et al., 2016; Malojcic et al., 2017; Li et al., 2018; Liu et al., 2020; Yu et al., 2020). For instance, with the developed three dimensional arterial spin labeling (3D-ASL) technique, Sun and colleagues found (Sun et al., 2016) widespread lower cerebral blood flow (CBF) in patients with symptomatic SVD in comparison to patients with non-symptomatic SVD, particularly where deficits in brain perfusion in the temporal and frontal lobe, hippocampus, thalamus, and insula were related to the degree of cognitive impairment. Reduced CBF, impaired cerebral autoregulation, and increased blood–brain barrier permeability were also manifested in subcortical areas of patients with SVD (Li et al., 2018). Region-specific malregulation of CBF has been suggested as a critical factor in SVD-related dementia, which may be linked to the progression of cognitive decline and hence used to track the course of disease progression (Shi et al., 2016; Malojcic et al., 2017). Moreover, in addition to lower perfusion-related cortical atrophy often reported in SVD, Schuff et al. (2009) observed a volumetric increase in subcortical WMH associated with reduced CBF in the frontal cortex. Meanwhile, Yu et al. (2020) reported a tight correlation of total SVD burden score (composed of lacunes, cerebral microbleeds, and enlarged perivascular spaces) with both global and regional CBF. Diffusion tensor imaging (DTI) is a sensitive technique to detect subtle changes of WM microstructural integrity, researchers have found that cognitive disturbances in subjects with SVD were related to abnormalities of multiple WM fibers connecting different cortical and subcortical regions (Tuladhar et al., 2015; Liu et al., 2020). It has been postulated that long-term hypoperfusion contributes to impairment of WM integrity, thereby leading to subcortical–cortical and cortical–cortical dysconnectivity, which is linked to diverse cognitive domains, namely “disconnection syndrome” (O'Sullivan et al., 2004). The disconnection of frontal–subcortical circuits is believed to be the underlying mechanism of cognitive impairment in SVD (O'Sullivan et al., 2001; Pantoni, 2010). However, whether and how cortical perfusion and WM damage jointly contribute to the early stage of cognitive impairment in patients with SVD remains unclear, which holds great implication for disease prevention and treatment.

To this end, we developed a cross-modal multimode imaging-based machine learning approach to investigate both diffusion and perfusion disturbances in a cohort of 113 patients with SVD, of which 74 were SVD patients with vMCI. We conducted a comprehensive battery of neuropsychological tests including attention, executive function, language, and working memory tests, and collected both DTI and ASL data from all subjects. From the imaging data, we extracted WM diffusion and cortical perfusion features including mean fractional anisotropy (FA), mean diffusivity (MD), axial diffusivity (AD), radial diffusivity (RD), and CBF within multiple regions of interest (ROIs) defined according to widely used gray and WM templates. Using diffusion-based, perfusion-based, and combined diffusion-perfusion features, we trained three sets of sparse logistic regression (SLR) classifiers to distinguish patients with vMCI from patients with normal cognition (control patients). Classification accuracy was evaluated using leave-one-out cross validation (LOOCV) and statistical comparisons were made between the three classifiers. Furthermore, we used the partial correlations to examine associations between the identified discriminative features and cognitive functions. Our research objective was to characterize abnormalities in gray matter perfusion and WM integrity, and enhance the understanding of the pathological evolution of cognitive decline in patients with SVD.

## Materials and Methods

### Participants

One hundred and thirteen patients with SVD were recruited from the Department of Neurology at RenJi Hospital between August 2017 and January 2020. SVD can be defined as subcortical WM hyperintensity on T2-weighted images with at least one lacunar infarct, following the criteria suggested by Galluzzi et al. (2005). Each subject underwent a standard evaluation, including neurological examination, complete sociodemographic and clinical data, and MRI examination. The inclusion criteria were as follows: (1) at least 6 years for education; (2) age 50–85 years; (3) informed consent form signed by the participant (Galluzzi et al., 2005). The following exclusion criteria were applied: (1) cortical and/or cortico-subcortical non-lacunar territorial infarcts and watershed infarcts; (2) neurodegenerative diseases (including Parkinson's disease and Alzheimer's disease); (3) signs of normal pressure hydrocephalus; (4) specific causes of WMH (e.g., metabolic, toxic, infectious, multiple sclerosis, brain irradiation); (5) alcoholic encephalopathy or illicit drug use; (6) major depression [Hamilton Depression Rating Scale (HDRS) ≥18]; (7) severe cognitive impairment (inability to perform the neuropsychological test or undergo the whole MRI scan); (8) MRI safety contraindications and claustrophobia (Galluzzi et al., 2005). All patients underwent laboratory examinations to exclude systemic or other neurological diseases.

### Neuropsychological Assessment

Neuropsychological assessments were performed within 1 week of the MRI examination. No patients suffered any transient ischemic attacks or strokes between the MRI examination and the evaluation. The Montreal Cognitive Assessment (MoCA) and Mini-Mental State Examination (MMSE) were used to assess overall cognitive performance. Moreover, a comprehensive battery of neuropsychological tests was designed to evaluate four key cognitive domains as described in previous studies (Hachinski et al., 2006; Xu et al., 2014). These tests were as follows: (1) attention and executive function: Trail-Making Tests A and B (TMT-A and TMT-B), Stroop color-word test (Stroop C-T), and verbal fluency test (VFT); (2) visuospatial function: Rey-Osterrieth Complex Figure Test (copy); (3) language function: Boston Naming Test (30 items); (4) memory function: auditory verbal learning test (short and long delayed free recall). Functional ability was assessed using the Katz basic activities of daily living (BADL) and Lawton and Brody instrumental activities of daily living (IADL) scales. The norms used here were based on mean scores of each measurement from a sample of typical elderly community members in Shanghai, China (Guo et al., 2007). Cognitive impairment was defined as 1.5 standard deviations below the normative mean on any neuropsychological test. The diagnostic criteria of vMCI included: (1) subjective cognitive difficulty reported by the patient or caregiver; (2) quantifiable cognitive decline within one or more cognitive domains (e.g., attention-executive function, memory, language, or visuospatial function); (3) normal instrumental activity of daily living. Controls were defined as SVD with no cognitive impairment, which means the scores of patients in all neuropsychological tests were within the normal range. After checking for the high quality of clinical and imaging data of enrolled participants, 74 vMCI participants and 39 age-, sex-, and education- matched controls were finally included in this study.

### MRI Acquisition

All MRI data were obtained using a 3.0 T MRI scanner (Signa HDxt; GE HealthCare, Milwaukee, WI, USA) equipped with an eight-channel phased array head coil. The following whole-brain sequences were obtained: (1) The sagittal T1-weighted images covering the whole brain were acquired by the 3D-fast spoiled gradient recalled echo (SPGR) sequence [repetition time (TR) = 5.6 ms, echo time (TE) = 1.8 ms, inversion time (TI) = 450 ms, flip angle = 15°, slice thickness = 1.0 mm, number of slices = 156, gap = 0, field of view (FOV) = 256 × 256 mm, and matrix = 256 × 256, scanning time=3′53′′]; (2) T2-fluid attenuated inversion recovery (FLAIR) sequences (TR = 9,075 ms, TE = 150 ms, TI = 2,250 ms, FOV = 256 × 256 mm, matrix = 256 × 256, slice thickness = 2 mm, and number of slices = 66, scanning time=7′18′′); (3) DTI (TR = 17,000 ms, TE = 89.8 ms, slice thickness = 2 mm, gap = 0, FOV = 256 × 256 mm, number of slices = 66, matrix = 128 × 128, and 20 diffusion-weighted directions with *b*-value = 1,000 s/mm^2^, scanning time = 6′14′′); (4) Pseudocontinuous ASL (pCASL) images were acquired using 3D fast spin-echo acquisition with background suppression and with a labeling duration of 1,500 ms and a post labeling delay of 2,000 ms, one control and one labeled images were acquired (TR = 4,337 ms, TE = 9.8 ms, FOV = 240 × 240 mm, slice thickness = 4 mm, flip angle = 155°, NEX = 3, and number of slices = 34 scanning time = 4′12′′). The total scanning time is 21′39′′.

### MRI Data Preprocessing

Processing of the diffusion MRI dataset was implemented using a pipeline toolbox, PANDA v1.3.1 (https://www.nitrc.org/ projects/panda), which is based on FMRIB's Software Library (FSL) tools. In the pipeline, skull-stripping with the brain extraction tool (BET) was done to extract brain tissue for b0 image in each subject. Eddy current-induced distortion and head motion artifacts were corrected by registering each raw diffusion-weighted image to the b0 image with an affine transformation. Diffusion metrics including FA, MD, AD, and RD were calculated within a mask created from b0 image. ASL images were post-processed at a General Electric Company (GE) workstation, version 4.4. ASL images of each subject were inspected for the excessive head movement (≥2 mm or 2°), and the area outside of the brain was excluded, then the quantitative CBF map of each subject was calculated.

The image registration was performed using Advanced Normalization Tools (ANTs) (http://stnava.github.io/ANTs/). The Johns Hopkins University International Consortium for Brain Mapping (ICBM)-DTI-81 FA template (Mori et al., 2008) was registered to the FA map of each individual using ANTs deformable registration. This transformation was inversed to warp the labels of WM regions in Johns Hopkins University ICBM atlas to individual FA space through General Label interpolation (WM regions listed in Supplementary Table 1). Quality control was performed through visual inspection of the FA map of each subject and the wrapped atlas in individual space. The CBF maps were skull-stripped by FSL with manual correction and then registered to 3D-T1WI structure imaging, the 3D-T1WI images were used for image registration and normalization into a standardized space that is consistent with the AAL template, with a reslicing resolution of 2 × 2 × 2 mm^3^. Mean values of diffusion parameter maps for each WM label were extracted. Moreover, the mean CBF value of GM labels in the AAL template was obtained. A total of 308 features, including 192 diffusion features and 116 CBF features, were extracted for each individual.

### Feature Selection

A sparse logistic regression classifier (Yamashita et al., 2008) with LOOCV was implemented to distinguish patients with vMCI from patients with SVD with normal cognition (control) using the combined features from the CBF and diffusion metrics. The workflow for the SLR-based classification framework is shown in Supplementary Figure R1. Before constructing the SLR classification model, it is necessary to determine a subset of discriminative features and elimination of the non-informative features for use in classification, which was widely employed to boost classification performance (Yahata et al., 2016; Drysdale et al., 2017). The standard lasso (Tibshirani, 1996) with a 10 × 10 nested feature selection (FS) method was employed to achieve a sparse model by excluding the majority of features from the model. Then, the SLR classifier was implemented on the basis of the optimal features. Concretely, the whole data set was split into 10-folds using a stratified approach, to keep an equal amount of (diagnosis and gender) combinations per fold. In each LOOCV fold, all-but-one subjects were used to train a SLR classifier, while the remaining subject was used for evaluation. Prior to LOOCV, the 10 × 10 nested FS was performed using lasso. In this way, the lasso was trained on different subsamples of the data set, to increase the stability of the selected features. The “test set” of the outer loop FS process was kept as a testing pool for LOOCV, whereas the 10-folds of the inner loop FS were used to select features. Consequently, the LOOCV folds that belonged to the same testing pool of the outer loop FS shared the same reduced features. In the inner loop FS, the FS was completed using *Statistics and regression Toolbox* of MATLAB (Mathworks Inc. version 2014a). Features were selected using the default setting of the lasso function. The hyperparameter λ was estimated default by lasso. The features selected at each inner fold and λ were combined by the union operation, to include features that are important for any possible subsample (inner 10 folds) of the training data set. Once the inner loop FS was executed, one participant was taken from the testing pool of the outer loop FS, and used as the test set of the LOOCV. The remaining samples were used to train SLR on the features retained during the inner loop FS.

Feature selection in each fold of the outer LOOCV was implemented using a slightly different sample subset, which led to a different set of selected features across folds. The “consensus” features that were selected on 75% folds of the outer LOOCV were defined as the discriminative features.

### Sparse Logistic Regression Classification

**Figure 1:**
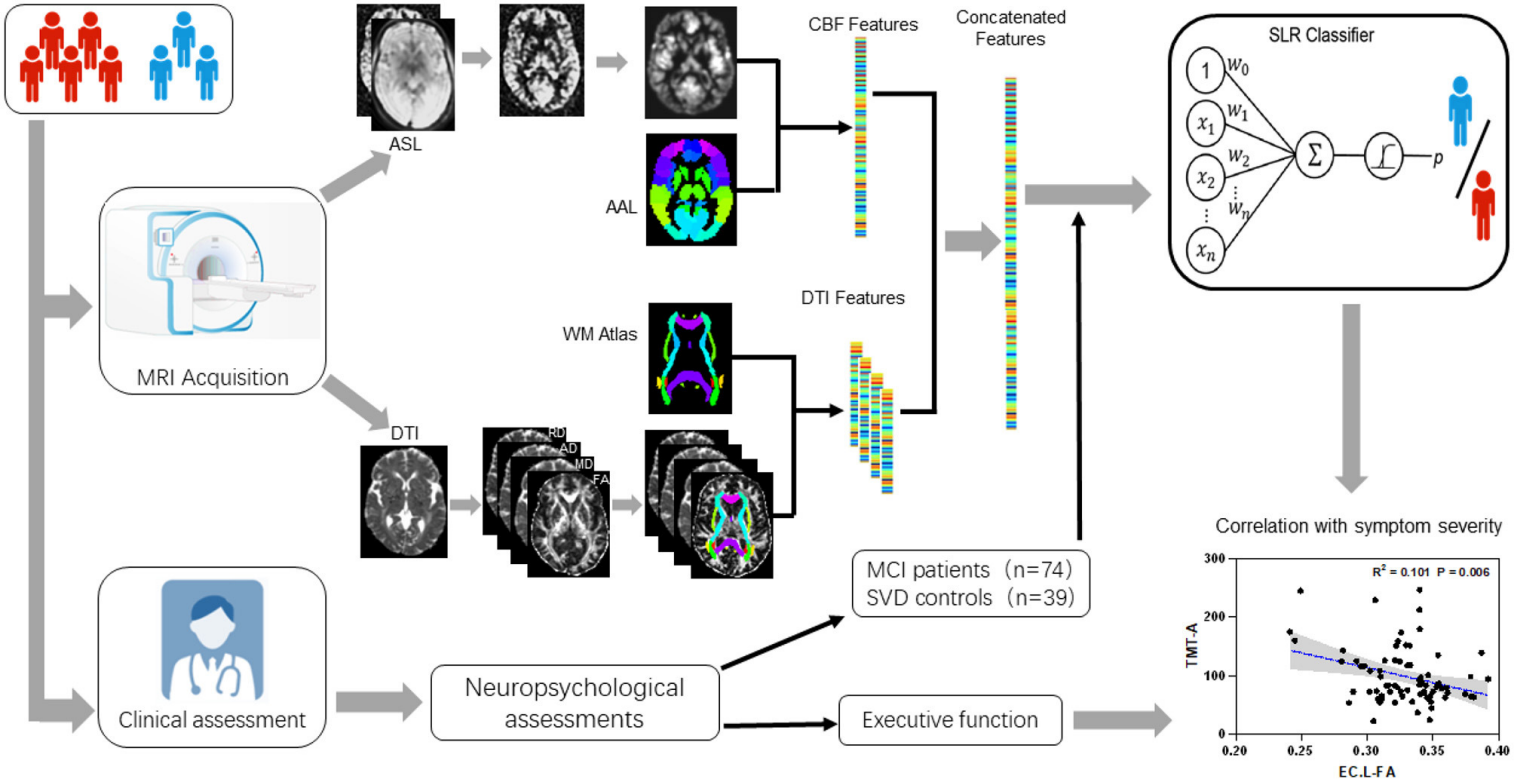
Research flow chart including: MRI procedures from DTI and ASL data collecting to features extracting, multimode imaging features including mean FA/MD/AD/RD and CBF within multiple ROIs defined by widely used ICBM templates for white matter diffusion features and AAL templates for gray matter perfusion features; A comprehensive battery of neuropsychological tests including general cognitive function, attention-executive function, visuospatial function, language and working memory, functional ability; Three sets of sparse logistic regression (SLR) classifiers were trained using diffusion-based, perfusion-based, and diffusion-perfusion combined features; Correlation of the final selected features with executive function.

To predict the diagnostic label from the optimal features, we employed logistic regression as the classifier. In logistic regression, a logistic function is used to define the probability of a participant belonging to the vMCI class as follows:

$$\begin{array}{l} P\left(y=1ẑ;w\right)=\frac{1}{1+\exp\left({-w}^{T}ẑ\right)} \end{array}$$

where *y* represents the diagnosis class label, that is *y* = 1 indicates patients with vMCI and *y* = 0 indicates patients with SVD with normal cognition (control), respectively. ẑ = [*z*^*T*^, 1]*T* ∈ ℝ^*k*+1^ is a feature vector with an augmented input. *w* ∈ ℝ^*k*+1^ is the weight vector of the logistic function. A receiver operating characteristic (ROC) curve was plotted to illustrate the classification ability of the model at varying discrimination thresholds. The predictive accuracy means the proportion of subjects who were correctly classified as a vMCI or a control label. To compare the ability of these classifiers to identify patients with vMCI, we applied the McNemar's test for comparing the area under the curve (AUC) of paired ROC curves (McNemar, 1947). The research flow chart is illustrated as Figure 1.

### Statistical Analysis

All data analyses and statistics were performed using R-3.6.0 (https://www.r-project.org). The Kolmogorov-Smirnov test was used to test the distribution of age, education, and identified features. Standard distribution data were compared using the *t*-test, and non-normally distributed data were analyzed using the Wilcoxon rank-sum test. A Chi-square test was used to compare the gender between the training set and the validation set. Partial correlations of Pearson were used to assess the associations between the identified imaging features and the scores of attention-executive function tests independently in vMCI and control groups, with sex, age, and education controlled as covariates. False discovery rate (FDR) was used for multiple comparison corrections.

## Results

### Demographic and Cognitive Characteristics

The demographic and cognitive characteristics of the participants are presented in Table 1. No significant differences in age, sex, and education were observed between the vMCI and the control patient groups. The mean MoCA score of the vMCI group was significantly lower than that of the control group (*p* < 0.01), with 85.14% of the patients with vMCI exhibiting executive dysfunction. The completion time for the TMT-A and TMT-B and the reaction time in the Stroop C-T test were significantly longer in the vMCI group than in the control group (all *p* < 0.01). The VFT performance was markedly worse in the vMCI group than in the control group (*p* < 0.01).

**Table 1:** Demographic and executive function characteristics.

	**vMCI**	**Controls**	***p-*value**
Number	74	39	
Age (y)	65.97 ± 6.84 (50–80)	63.44 ± 7.04 (52–81)	0.066
Male (%)	57 (77.03%)	30 (76.9%)	0.610
Education (y)	10.51 ± 2.69	10.54 ± 2.47	0.962
MoCA	21.72 ± 3.43	26.33 ± 1.23	<0.001
MMSE	27.17 ± 1.98	28.49 ± 1.23	<0.001
TMT-A	99.18 ± 50.60	59.46 ± 15.10	<0.001
TMT-B	225.38 ± 87.38	150.83 ± 38.41	<0.001
Stroop C-T	126.40 ± 56.94	79.26 ± 15.36	<0.001
VFT	13.01 ± 4.00	16.12 ± 3.63	<0.001

*Data represent means ± standard deviation, with the range in parentheses, if applicable. vMCI, subcortical vascular mild cognitive impairment; TMT-A, trail-making tests A; TMT-B, trail-making tests B; Stroop C-T, stroop color-word test; VFT, verbal fluency test*.

### Diffusion and Perfusion Features Predicted Vascular Mild Cognitive Impairment Patients

**Figure 2:**
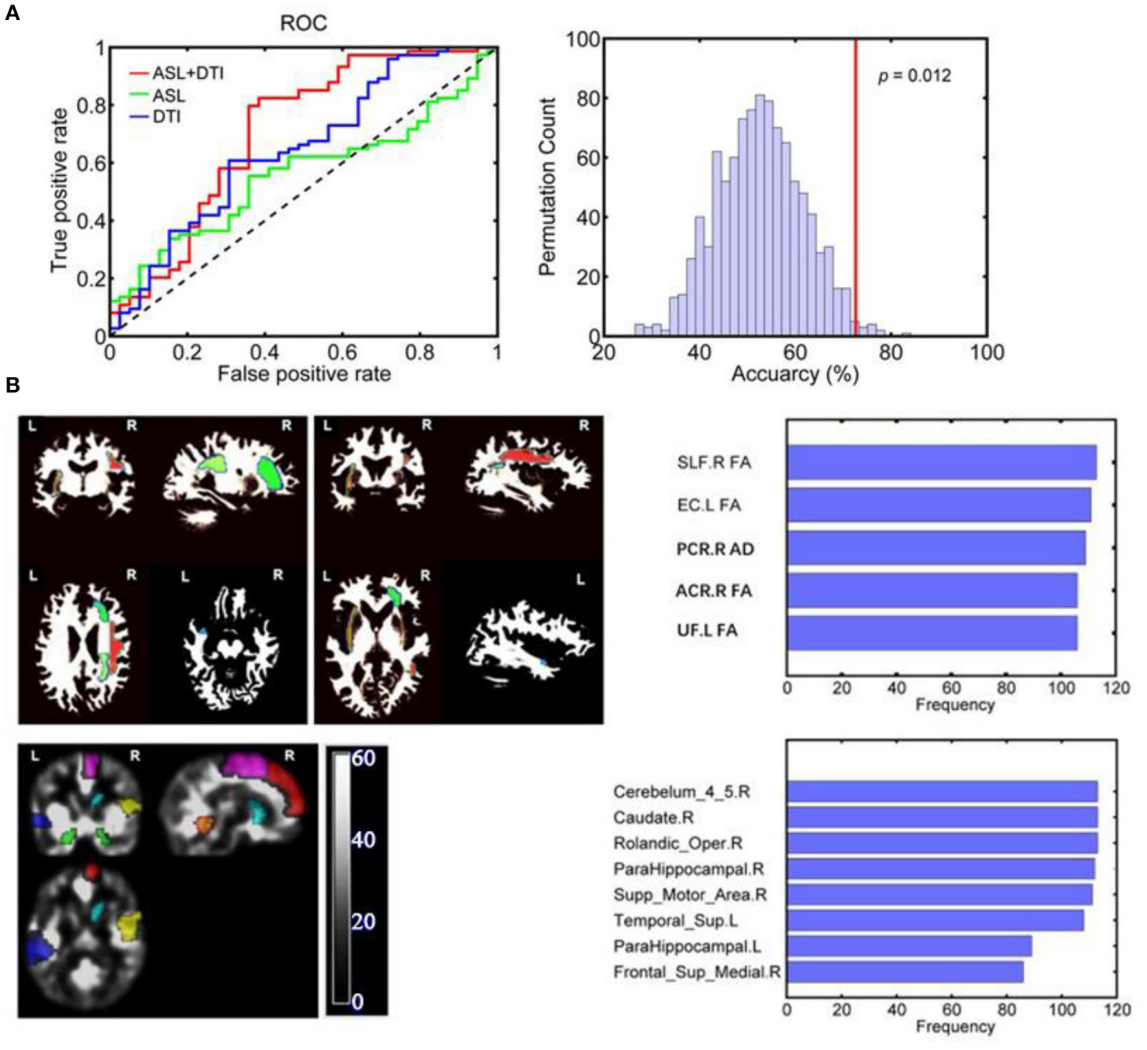
**(A)** ROC curves of each SLR classifier for discriminating vMCI and controls: The AUC for the combined model, a single ASL model, and a single DTI model were 0.708, 0.559, and 0.647, respectively. **(B)** The discriminative gray and white matter regions for SLR classifier based on combined features. The combined CBF areas included Rolandic_oper, Supp_Motor_Area, Frontal_Sup_Medial, ParaHippocampal and Caudate of the right hemisphere as well as ParaHippocampal and Temporal_ Sup in the left hemisphere. The combined DTI features included ACR_FA, PCR_AD, and SLF_FA of the right hemisphere as well as EC_FA and UF_FA in the left hemisphere. SLF, superior longitudinal fasciculus; EC, external capsule; ACR, anterior corona radiata; PCR, posterior corona radiata; UF, uncinate fasciculus; L, left; R, right.

Distinct features with a frequency of ≥75% for distinguishing patients with vMCI from control patients were selected and used to construct SLR classifiers. The performance results of the SLR classifiers, including both single-mode models and a combined model with both diffusion and perfusion features, are shown in Figure 2A and Tables 2, 3. Compared with the single-mode models, the SLR classifier with both diffusion and perfusion features achieved the highest accuracy of 72.57%, with sensitivity of 77.03%.

**Table 2:** Classification performance of SLR classifiers using diffusion features, CBF features, and their combined features.

**Model**	**Accuracy (%)**	**Sensitivity (%)**	**Specificity (%)**	**AUC**	***p***
CBF+DTI	72.57	77.03	64.10	0.708	–
CBF	57.52	62.16	48.72	0.559	0.003
DTI	61.06	64.86	53.85	0.647	0.039

*AUC, area under curve. Compared to single model with diffusion/perfusion features (p = 0.003/p = 0.039), the SLR classifier achieved the highest accuracy using combined diffusion and perfusion features (accuracy 72.57%, sensitivity 77.03%)*.

**Table 3:** Identified combined diffusion/perfusion features for discriminating vMCI and Controls.

**Frequency**	**Type**	**Region**	**vMCI**	**Controls**	***p*-values**
1.000	CBF	Rolandic_Oper_R	49.215	55.617	0.020^*^
0.982	CBF	Supp_Motor_Area_R	41.087	48.511	0.003^*^
0.761	CBF	Frontal_Sup_Medial_R	37.611	40.417	0.162
0.788	CBF	ParaHippocampal_L	45.680	50.681	0.020^*^
0.991	CBF	ParaHippocampal_R	44.219	50.527	0.003^*^
1.000	CBF	Caudate_R	34.338	35.348	0.342
0.956	CBF	Temporal_Sup_L	51.629	56.769	0.094
1.000	CBF	Cerebelum_4_5_R	41.420	43.792	0.342
0.938	FA	Right anterior corona radiata	0.316	0.346	0.002^*^
0.965	AD^#^	Right posterior corona radiata	13.612	13.125	0.142
0.982	FA	Left external capsule	0.328	0.350	0.002^*^
1.000	FA	Right superior longitudinal fasciculus	0.369	0.380	0.162
0.938	FA	Left uncinate fasciculus	0.336	0.357	0.039^*^

# *Unit is 10-4;*

*a *p < 0.05 corrected by FDR. CBF, cerebral blood flow; FA, fractional anisotropy; AD, axial diffusivity; Rolandic_Oper, rolandic operculum; Supp_Motor_Area, supplementary motor area; Frontal_Sup_Medial, medial superior frontal gyrus; ParaHippocampal, parahippocampal; Temporal_Sup, superior temporal gyrus; FDR, false discovery rate; vMCI, subcortical vascular mild cognitive impairment*.

The classification results are shown as an ROC curve using each classification score of subject as a threshold in Figure 2A. The AUCs for the combined model, single ASL model, and single DTI model were 0.708, 0.559, and 0.647, respectively.

The CBF areas in the combined model included the right Rolandic operculum, supplementary motor area (SMA), medial superior frontal gyrus (mSFG), parahippocampal gyrus and caudate, and the left parahippocampal and superior temporal gyrus (STG). The DTI features in the combined model included the FA of the right anterior corona (ACR) radiata, right superior longitudinal fasciculus (SLF), left external capsule and left uncinate fasciculus, and the AD of the right posterior corona radiata (PCR). The combined CBF and DTI features are shown in Figure 2B.

### Associations Between Executive Function and Diffusion and Perfusion Features

**Figure 3:**
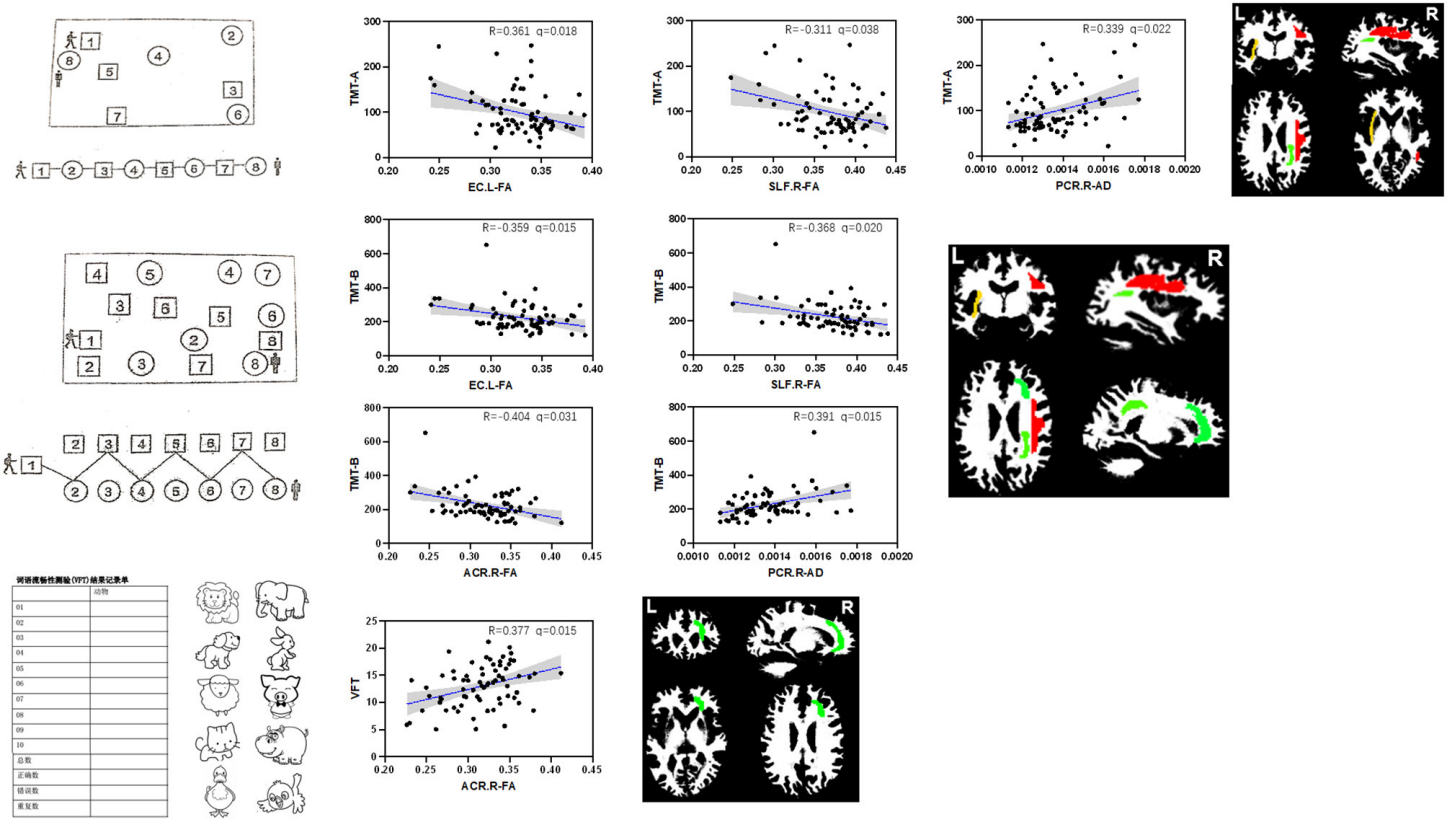
Correlations between discriminative features and executive function tests within vMCI group, controlled for gender, age, and education. All results were corrected by FDR. The mean AD value of right PCR (*r* = 0.339, *P* < 0.037) and the mean FA values of the left EC (*r* = −0.361, *P* < 0.026) were significantly associated with TMT-A; the mean FA values of the right ACR (*r* = −0.404, *P* < 0.026), left EC (*r* = −0.359, *P* < 0.026) and right SLF (*r* = −0.368, *P* < 0.026), and the mean AD value of right PCR (*r* = 0.391, *P* < 0.026) were significantly associated with TMT-B; the mean FA values of the right ACR (*r* =0.377, *P* < 0.026) were significantly associated with VFT.

In the vMCI group, correlation analysis showed that the mean AD of the right PCR (*r* = 0.339, *p* < 0.037) and the mean FA of the left external capsule (*r* = −0.361, *p* < 0.026) were significantly associated with TMT-A time. The mean FA of the right ACR (*r* = −0.404, *p* < 0.026), left external capsule (*r* = −0.359, *p* < 0.026), and right SLF (*r* = −0.368, *p* < 0.026), and the mean AD of the right PCR (*r* = 0.391, *p* < 0.026), were significantly associated with the TMT-B time. The mean FA of the right ACR (*r* =0.377, *p* < 0.026) was significantly associated with VFT, as shown in Figure 3 and Table 4. No discriminative perfusion feature showed a significant association with attention-executive performance, as shown in Table 4. None of the discriminative perfusion and diffusion features were significantly associated with attention-executive performance within control group, as shown in Supplementary Table 2.

**Table 4:** Correlations between discriminative combined diffusion/perfusion features and executive function tests in vMCI group.

**Type**	**Region**	**TMT-A**		**TMT-B**		**Stroop C-T**		**VFT**	
		**R**	***p***	**R**	***p***	**R**	***p***	**R**	***p***
CBF	Rolandic_Oper_R	0.003	0.592	0.103	0.407	−0.043	0.534	0.058	0.522
CBF	Supp_Motor_Area_R	−0.183	0.242	0.037	0.547	0.052	0.522	0.144	0.293
CBF	Frontal_Sup_Medial_R	0.066	0.510	0.007	0.587	−0.002	0.582	0.140	0.289
CBF	ParaHippocampal_L	−0.190	0.228	−0.049	0.519	0.014	0.582	0.163	0.268
CBF	ParaHippocampal_R	−0.161	0.263	−0.024	0.580	0.100	0.394	0.139	0.282
CBF	Caudate_R	0.055	0.522	0.237	0.187	−0.084	0.456	−0.009	0.593
CBF	Temporal_Sup_L	−0.102	0.398	0.016	0.588	−0.031	0.560	0.146	0.300
CBF	Cerebelum_4_5_R	−0.160	0.256	−0.169	0.269	0.017	0.595	0.233	0.184
FA	Right anterior corona radiata	−0.216	0.166	−0.404	0.031^*^	−0.181	0.238	0.377	0.015^*^
AD^#^	Right posterior corona radiata	0.339	0.022^*^	0.391	0.015^*^	0.209	0.177	−0.219	0.184
FA	Left external capsule	−0.361	0.018^*^	−0.359	0.015^*^	−0.168	0.260	0.313	0.173
FA	Right superior longitudinal fasciculus	−0.311	0.042^*^	−0.368	0.020^*^	−0.219	0.200	0.221	0.207
FA	Left uncinate fasciculus	−0.061	0.522	−0.141	0.295	−0.137	0.280	0.070	0.504

# *Unit is 10^−4^;*

* *p < 0.05, corrected by FDR. The selected white matter diffusion features were significantly associated with TMT-A/TMT-B/VFT. No discriminative perfusion feature was detected associated with attention-executive performance significantly*.

## Discussion

Associations between cognitive decline and impairments to anterior thalamic radiation (ACR) have been broadly reported in SVD. Voxel-based lesion-symptom mapping studies (Duering et al., 2011, 2014; Biesbroek et al., 2013) found that strategic locations of WM damage within ACR were associated with processing speed performance or executive function in SVD. Tract-based spatial statistics study also found that diffusion metrics along the forceps minor and ACR were discriminative for cognitive impairments in patients with SVD (Chen et al., 2018), which is consistent with the present finding that ACR diffusion abnormalities not only contributed to the classification accuracy of patients with SVD with-/without- cognitive symptoms, but also were significantly correlated with executive function. This indicates the involvement of the ACR in the early stage of cognitive decline in SVD. Furthermore, other discriminative WM fibers revealed in our study constituted the lateral pathway of the cholinergic system (external capsule, uncinate fasciculus, CR, and SLF), which radiates to the dorsal frontoparietal neocortex, the temporal cortex, and the parahippocampal gyrus (Caruso et al., 2019; Nolze-Charron et al., 2020). Specifically, fiber bundles that radiated to the dorsal frontoparietal cortex were associated with performance in the executive function tests in the vMCI group. As a matter of a fact, cholinergic dysregulation in SVD has been discussed extensively, including cholinergic neuronal deficits and cholinergic denervation (Mesulam et al., 2003; Keverne et al., 2007), decreased cerebrospinal fluid acetylcholine concentrations (Wallin et al., 2003), and the promising effects of cholinergic therapies (Caruso et al., 2019). In particular, a tractography study (Liu et al., 2017) identified significantly lower FA within cholinergic pathways (including the external capsule, cingulum, and claustrum) in patients with vascular cognitive impairment no dementia group. The disrupted pathways could fully explain the executive dysfunction and partly explain the memory and global cognitive impairments. Another tractography study isolated the external capsule as the lateral cholinergic tract and found that diffusion metrics of both the external capsule and the overlying SLF were correlated with executive dysfunction (Nolze-Charron et al., 2020). Our findings are consistent with these reports, with a broad range of lateral cholinergic tracts up and down the external capsule being significantly related to executive dysfunction in the early stage of cognitive decline in SVD but not the non-symptomatic stage. Collectively, results that showed frontal fiber dysconnectivity and potential cholinergic dysregulation shed light on the clinical characteristics of attention and executive dysfunction in vMCI, thereby supporting a physically active lifestyle and cholinergic therapy as a potential effective treatment option for vMCI (Dey et al., 2016; Strömmer et al., 2020).

Cortical perfusion abnormalities in frontal (mSFG, SMA, Rolandic operculum), subcortical (caudate nucleus), and limbic (parahippocampal gyrus) areas also contributed to the accuracy of subtype classification in the present machine learning model, although no associations with performance of cognitive tests in these patients were found. Previous ASL studies showed widespread significant reductions in cortical CBF in patients with SVD with cognitive impairment (Schuff et al., 2009; Gao et al., 2013; Sun et al., 2016), although the spatial profiles of CBF abnormalities reported among these studies were rather divergent. Cortical perfusion is regulated by neurovascular coupling and a complex autoregulation system, and may not therefore be simply related to cognitive impairment (Caruso et al., 2019). Recent studies suggested an important role for the autonomic nervous system in the maintenance of CBF (Hamner et al., 2012). It was suggested that cholinesterase inhibitors modulate cerebral vascular functions because of the possible role of cholinergic fibers in cerebral flow regulation (Brown and Thore, 2011). Considering the WM diffusion abnormalities in our classification model, CBF disturbance of the frontal–subcortical–limbic system may partly result from dysfunction of the lateral capsular pathway of cholinergic tracts which needs further study. Moreover, recent study showed that cortical perfusion abnormalities may also affect cognition through secondary changes in subcortical myelin content (Chen et al., 2013; Bouhrara et al., 2020). The diffusion-perfusion combined classifier with the highest cognitive classification accuracy in this study might suggest the interaction of gray matter perfusion and WM integrity, which explained the cognitive outcomes.

This study had several limitations. First, because of inherent limitations of the atlas used for WM parcellation, only the main WM tracts were evaluated, and fibers in superficial regions were not included in our study. Future studies of the fibers in superficial regions may provide additional information on vMCI. Second, the low spatial resolution of the CBF images may have resulted in partial volume effects causing bias in the CBF features. Third, the results were not validated on an external dataset. Further studies using multicenter validation datasets are needed to acquire high-level evidence. Fourth, resting CBF only provides information for a cut-off time point, at which CBF might still be relatively preserved or compensated. Fifth, although detailed clinical history, imaging analysis, and neuropsychological evaluation were used to avoid the interference of AD, the influence of mixed dementia on this study could not be completely excluded. Finally, compared with the dimensionality of the features, the sample size was relatively small. In addition, there were more males in both groups, which may lead to biasness in results.

## Conclusions

We demonstrated the superior accuracy of using diffusion-perfusion combined multimode imaging features for classifying vMCI subtype out of a cohort of patients with SVD. Importantly, these findings highlight that disrupted WM integrity might play a critical role in the progression of cognitive impairment in patients with SVD, while malregulation of coritcal perfusion needs further study.

## Data Availability Statement

The raw data supporting the conclusions of this article will be made available by the authors, without undue reservation.

## Ethics Statement

The studies involving human participants were reviewed and approved by Research Ethics Committee of Renji Hospital, School of Medicine, Shanghai Jiao Tong University. The patients/participants provided their written informed consent to participate in this study.

## Author Contributions

YW: data collection, image registration, statistical analysis, data interpretation, and manuscript preparation. PL: data collection and neuropsychological assessment. YZha: statistical analysis, data interpretation, and manuscript preparation. XW: data collection and image registration. YQ: data collection. ZW: data interpretation and manuscript preparation. QX: study design. YZho: study design and commenting on drafts. All authors contributed to the article and approved the submitted version.

## Conflict of Interest

The authors declare that the research was conducted in the absence of any commercial or financial relationships that could be construed as a potential conflict of interest.

## Publisher's Note

All claims expressed in this article are solely those of the authors and do not necessarily represent those of their affiliated organizations, or those of the publisher, the editors and the reviewers. Any product that may be evaluated in this article, or claim that may be made by its manufacturer, is not guaranteed or endorsed by the publisher.
