# Supplementary material for: The Contribution of White Matter Diffusion and Cortical Perfusion Pathology to Vascular Cognitive Impairment: A Multimode Imaging-Based Machine Learning Study
Source: Front Aging Neurosci. 2021 Aug 6;13:687001. doi: 10.3389/fnagi.2021.687001 (PMC8379092; doi:10.3389/fnagi.2021.687001)
Supplement: Supplementary file 1 [file Data_Sheet_1.docx]

**Table S1** Labeled Regions in JHU ICBM-DTI-81 White-Matter Atlas

| number | White matter label name |
| --- | --- |
| 1 | Middle cerebellar peduncle |
| 2 | Pontine crossing tract (a part of middle cerebellar peduncle) |
| 3 | Genu of corpus callosum |
| 4 | Body of corpus callosum |
| 5 | Splenium of corpus callosum |
| 6 | Fornix (column and body of fornix) |
| 7 | Right corticospinal tract |
| 8 | Left corticospinal tract |
| 9 | Right medial lemniscus |
| 10 | Left medial lemniscus |
| 11 | Right inferior cerebellar peduncle |
| 12 | Left inferior cerebellar peduncle Page 6 of 6 |
| 13 | Right superior cerebellar peduncle |
| 14 | Left superior cerebellar peduncle |
| 15 | Right cerebral peduncle |
| 16 | Left cerebral peduncle |
| 17 | Right anterior limb of internal capsule |
| 18 | Left anterior limb of internal capsule |
| 19 | Right posterior limb of internal capsule |
| 20 | Left posterior limb of internal capsule |
| 21 | Right retrolenticular part of internal capsule |
| 22 | Left retrolenticular part of internal capsule |
| 23 | Right anterior corona radiata |
| 24 | Left anterior corona radiata |
| 25 | Right superior corona radiata |
| 26 | Left superior corona radiata |
| 27 | Right posterior corona radiata |
| 28 | Left posterior corona radiata |
| 29 | Right posterior thalamic radiation (include optic radiation) |
| 30 | Left posterior thalamic radiation (include optic radiation) |
| 31 | Right sagittal stratum(include inferior longitidinal fasciculus and inferior frontooccipital fasciculus) |
| 32 | Left sagittal stratum (include inferior longitidinal fasciculus and inferior frontooccipital fasciculus) |
| 33 | Right external capsule |
| 34 | Left external capsule |
| 35 | Right cingulum (cingulate gyrus) |
| 36 | Left cingulum (cingulate gyrus) |
| 37 | Right cingulum (hippocampus) |
| 38 | Left cingulum (hippocampus) |
| 39 | Right fornix (cres)/Stria terminalis (cannot be resolved with current resolution) |
| 40 | Left fornix (cres)/Stria terminalis (cannot be resolved with current resolution) |
| 41 | Right superior longitudinal fasciculus |
| 42 | Left superior longitudinal fasciculus |
| 43 | Right superior fronto-occipital fasciculus (could be a part of anterior internal capsule) |
| 44 | Left superior fronto-occipital fasciculus (could be a part of anterior internal capsule) |
| 45 | Right uncinate fasciculus |
| 46 | Left uncinate fasciculus |
| 47 | Right tapetum |
| 48 | Left tapetum |

**Table S2** Correlations between discriminative combined diffusion/perfusion features and executive function tests in control group

| Type | region | TMT-A | | TMT-B | | Stroop C-T | | VFT | |
| --- | --- | --- | --- | --- | --- | --- | --- | --- | --- |
|  |  | r | P | r | P | r | P | r | P |
| CBF | Rolandic_Oper_R | 0.017 | 0.945 | -0.333 | 0.376 | -0.104 | 0.789 | -0.096 | 0.789 |
| CBF | Supp_Motor_Area_R | -0.053 | 0.890 | -0.176 | 0.700 | -0.229 | 0.647 | -0.173 | 0.700 |
| CBF | Frontal_Sup_Medial_R | -0.055 | 0.890 | -0.245 | 0.604 | -0.139 | 0.789 | -0.170 | 0.700 |
| CBF | ParaHippocampal_L | 0.046 | 0.896 | -0.295 | 0.493 | -0.124 | 0.789 | -0.255 | 0.604 |
| CBF | ParaHippocampal_R | -0.114 | 0.789 | -0.445 | 0.173 | -0.133 | 0.789 | -0.186 | 0.700 |
| CBF | Caudate_R | -0.415 | 0.173 | -0.408 | 0.173 | -0.085 | 0.810 | -0.094 | 0.789 |
| CBF | Temporal_Sup_L | 0.051 | 0.890 | -0.264 | 0.591 | -0.298 | 0.493 | -0.109 | 0.789 |
| CBF | Cerebelum_4_5_R | -0.242 | 0.604 | -0.429 | 0.173 | -0.077 | 0.831 | -0.119 | 0.789 |
| FA | Right anterior corona radiata | 0.406 | 0.173 | 0.198 | 0.700 | 0.183 | 0.700 | -0.034 | 0.913 |
| AD^#^ | Right posterior corona radiata | 0.415 | 0.173 | 0.404 | 0.173 | 0.095 | 0.789 | -0.039 | 0.909 |
| FA | Left external capsule | -0.265 | 0.591 | 0.028 | 0.919 | -0.151 | 0.763 | 0.174 | 0.700 |
| FA | Right superior longitudinal fasciculus | -0.350 | 0.334 | -0.167 | 0.700 | -0.187 | 0.700 | -0.013 | 0.945 |
| FA | Left uncinate fasciculus | -0.440 | 0.173 | -0.193 | 0.700 | 0.111 | 0.789 | 0.108 | 0.789 |

Note:#Unit is 10^-4^, Note:*P < 0.05, corrected by FDR. None of the discriminative perfusion and diffusion features were significantly associated with attention-executive performance within control group


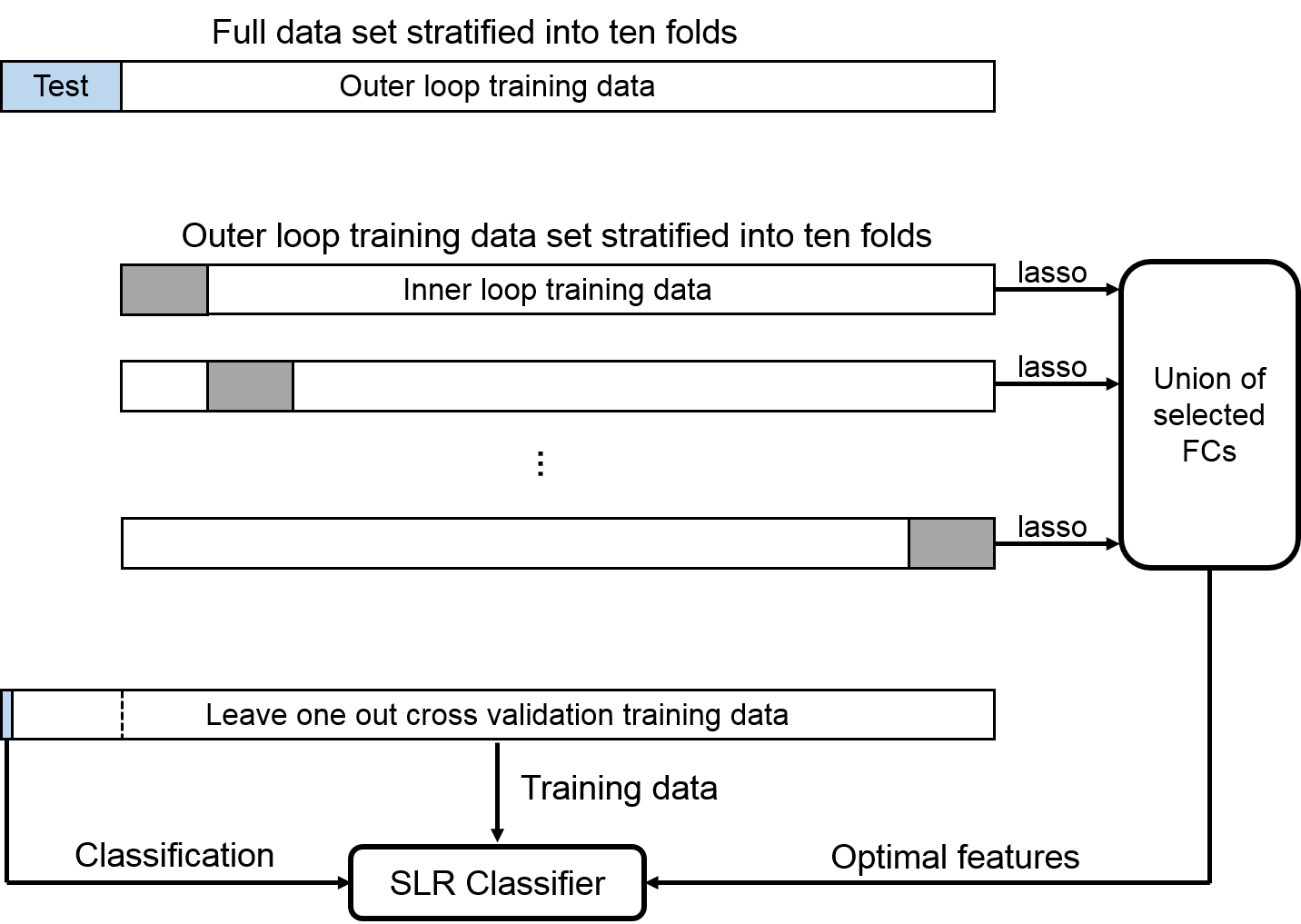


Figure R1. Illustration of nested 10×10 feature selection and leave-one-out cross-validation. The whole data set was stratified into ten folds: one was left out as the testing pool for LOOCV, and the remaining 9 folds (outer loop training data) were used for constructing the optimal features. Inner feature selection loop: The outer loop training data was stratified into ten folds: one was left out, and the remaining 9 folds (inner loop training data) were subject to lasso feature selection. Optimal features were the union of the functional connections (FCs) selected throughout the inner loop. Outer LOOCV predicting loop: In each LOOCV fold, one sample was taken from the testing pool of the outer loop and used as a test set for evaluation. The remaining samples were used to train a SLR classifier on optimal features retained during the inner training loop. This procedure is repeated for every sample in the testing pool of the outer loop.

**Table R1** Demographic in discovery dataset and holdout dataset.

|  | Discovery dataset | Holdout dataset | P |
| --- | --- | --- | --- |
| Age | 64.84±7.16 | 65.75±6.58 | 0.535 |
| Diagnostic label (vMCI/control patient) | 52/29 | 22/10 | 0.647 |
